# Application of diffusion kurtosis tensor MR imaging in characterization of renal cell carcinomas with different pathological types and grades

**DOI:** 10.1186/s40644-021-00394-7

**Published:** 2021-03-16

**Authors:** Jie Zhu, Xiaojie Luo, Jiayin Gao, Saying Li, Chunmei Li, Min Chen

**Affiliations:** Department of Radiology, Beijing Hospital, National Center of Gerontology, Institute of Geriatric Medicine, Chinese Academy of Medical Sciences, Beijing, P. R. China

**Keywords:** Diffusion kurtosis tensor imaging, Renal cell carcinoma, Subtype, Nuclear grade

## Abstract

**Background:**

To probe the feasibility and reproducibility of diffusion kurtosis tensor imaging (DKTI) in renal cell carcinoma (RCC) and to apply DKTI in distinguishing the subtypes of RCC and the grades of clear cell RCC (CCRCC).

**Methods:**

Thirty-eight patients with pathologically confirmed RCCs [CCRCC for 30 tumors, papillary RCC (PRCC) for 5 tumors and chromophobic RCC (CRCC) for 3 tumors] were involved in the study. Diffusion kurtosis tensor MR imaging were performed with 3 b-values (0, 500, 1000s/mm^2^) and 30 diffusion directions. The mean kurtosis (MK), axial kurtosis (Ka), radial kurtosis (Kr) values and mean diffusity (MD) for RCC and contralateral normal parenchyma were acquired. The inter-observer agreements of all DKTI metrics of contralateral renal cortex and medulla were evaluated using Bland-Altman plots. Statistical comparisons with DKTI metrics of 3 RCC subtypes and between low-grade (Furman grade I ~ II, 22 cases) and high-grade (Furman grade III ~ IV, 8 cases) CCRCC were performed with ANOVA test and Student *t* test separately. Receiver operating characteristic (ROC) curve analyses were used to compare the diagnostic efficacy of DKTI metrics for predicting nuclear grades of CCRCC. Correlations between DKTI metrics and nuclear grades were also evaluated with Spearman correlation analysis.

**Results:**

Inter-observer measurements for each metric showed great reproducibility with excellent ICCs ranging from 0.81 to 0.87. There were significant differences between the DKTI metrics of RCCs and contralateral renal parenchyma, also among the subtypes of RCC. MK and Ka values of CRCC were significantly higher than those of CCRCC and PRCC. Statistical difference of the MK, Ka, Kr and MD values were also obtained between CCRCC with high- and low-grades. MK values were more effective for distinguishing between low- and high- grade CCRCC (area under the ROC curve: 0.949). A threshold value of 0.851 permitted distinction with high sensitivity (90.9%) and specificity (87.5%).

**Conclusion:**

Our preliminary results suggest a possible role of DKTI in differentiating CRCC from CCRCC and PRCC. MK, the principle DKTI metric might be a surrogate biomarker to predict nuclear grades of CCRCC.

**Trial registration:**

ChiCTC, ChiCTR-DOD-17010833, Registered 10 March, 2017, retrospectively registered, http://www.chictr.org.cn/showproj.aspx?proj=17559.

## Background

Renal cell carcinoma (RCC) is the most common malignant renal tumor in adults. Although surgery is the most effective treatment, other options which are less aggressive approaches, including radiofrequency ablation, cytoablation and even active surveillance have been applied [1]. In advanced disease, systemic therapy with targeted agents, immunotherapy and chemotherapy were used to reduce the risk of recurrence and improve survival [2]. Thus, identification of subtypes and histologic grades prior to treatment has clinical significance in determining a treatment strategy and evaluating prognosis.

Diffusion weighted imaging (DWI) has been extensively explored to detect the histological characteristics of RCC for differential diagnosis, histologic subtyping, and defining the histologic grade [3, 4]. The principle of DWI is based on the assumption of a Gaussian distribution of displacement probabilities of water molecules due to water self-diffusion. However, the diffusion of water molecules is not Gaussian in most tissues of the human body because of the complex structures. Diffusion kurtosis imaging (DKI) based on non-Gaussian diffusion models might assess the complexity of microstructural environments more accurately than conventional DWI [5].

DKI was initially applied exclusively in brain imaging [6]. Recent studies had extended DKI to challenging applications in various extra-cranial regions [7–13] (e.g. breast, lung, liver, prostate, rectum and kidney). Sun et al. [8] showed that DKI was superior to DWI for assessment of benign and malignant breast lesions. Several studies demonstrated that, in compared with apparent diffusion coefficient (ADC), kurtosis from DKI had higher correlation with histologic grades of breast cancer [8], rectal adenocarcinomas [9], endometrial cancer [14] and RCC [13]. DKI was also successfully applied for differentiating the subtypes of rectal carcinoma [9]. In particular, DKI had shown potential for characterizing and evaluating the histological characteristic of lesions.

In previous studies of DKI applied in extra-cranial region, there are two common models to produce kurtosis images. Some studies [8–10] estimated kurtosis through trace-weighted images (TWIs) with 3 diffusion directions and a minimum of 3 b-values. Only mean kurtosis (MK) and mean diffusity (MD) were derived from this model. As kurtosis is a tensorial quantity, this straightforward estimation of kurtosis might introduce bias and error [15, 16]. Another model is the extension from diffusion tensor imaging (DTI) requiring the use of at least 3 b-values and 15 diffusion directions [7, 11, 12, 17]. It is also described as diffusion kurtosis tensor imaging (DKTI). DKTI-derived indices include kurtosis metrics, i.e. axial kurtosis (Ka) and radial kurtosis (Kr), in addition to MK. Conventional diffusion metrics, such as fractional anisotropy (FA) and MD were also involved.

The aim of this investigation was to probe the feasibility and characteristics of DKTI in RCC and to apply DKTI in distinguishing subtypes of RCC and the grades of clear cell RCC (CCRCC).

## Methods

### Patients

This study was approved by the local Institutional Review Board. All patients provided written informed consent before the study and consent to publish the medical images included in the Figures. Sixty patients suspected of renal solid mass based on previous CT or ultrasonography examinations were recruited into the prospective study to undergo renal MR examination including diffusion kurtosis tensor MR imaging. Some were excluded for the following reasons: 1) without surgery and histopathological results (*n* = 12); 2) histopathological results were not RCC, including angiomyolipoma (*n* = 6), oncocytoma (*n* = 1), renal cyst (*n* = 2) and collecting duct carcinoma (*n* = 1).

### MR examination

All patients in this study were performed on a 3.0-T MR scanner [GE DISCOVERY MR750 (GE Healthcare, Milwaukee, WI, USA) with 23/40 mT/m maximum gradient strength and 80/150 mT/m/s gradient slew rate] equipped with a four-channel torso phased array coil. For morphologic evaluation of the kidneys, the routine MRI protocol included a transverse breath-hold T1-weighted gradient-recalled echo with in-phase and out-of-phase sequences, a respiratory-triggered transverse and coronal T2-weighted fast spin-echo sequences with fat saturation, followed by transverse breath-hold single-shot spin-echo echo-planar DW imaging with 3 sets of b values (0, 200 and 1000 s/mm^2^).

DKTI was performed with a respiratory triggering single shot EPI sequence in the transverse plane with 3 sets of b values (0, 500 and 1000 s/mm^2^), 30 diffusion directions. The other imaging parameters were as follows: echo time (TE) = 55.7–58.9 ms, repetition time (TR) = 2500 ms, matrix = 128 × 128, field of view (FOV) = 380 mm, Bandwidth = 1953(Hz/pixel), 20 slices with a slice thickness of 5 mm, parallel acquisition with acceleration factor of 2, number of excitations (NEX) = 2. The mean acquisition time was 5:43 ± 0:28 min (range, 5:06–6:35 min).

### Imaging analysis

**Fig. 1:**
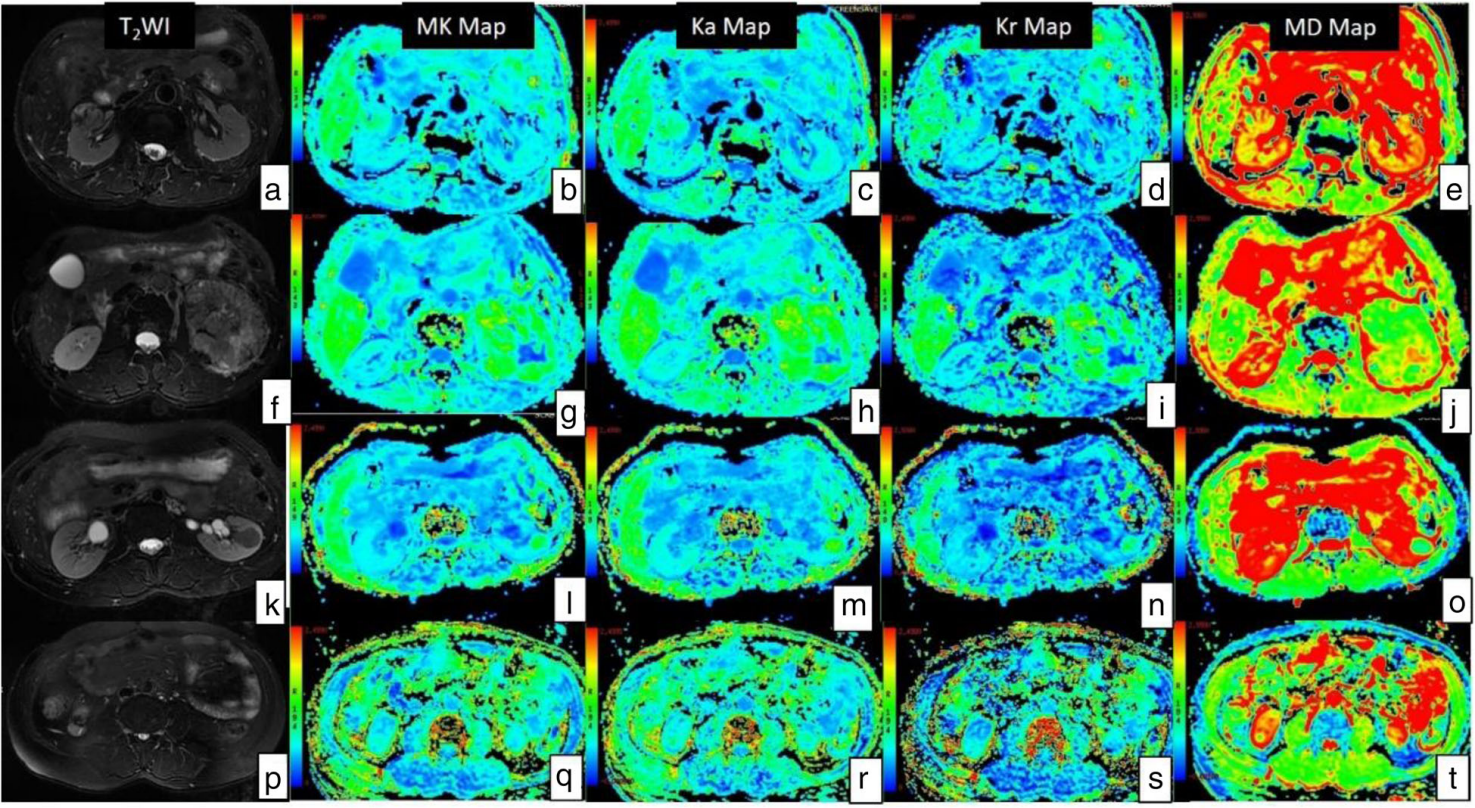
Images of 3 subtypes of RCC, including the low- and high- grade of CCRCC. T2WI, MK maps, Ka maps, Kr maps and MD maps of low-grade CCRCC (**a, b, c, d, e**), high-grade CCRCC (**f, g, h,i, j**), CRCC (**k, l, m, n, o**), PRCC (**p, q, r, s, t**). The color scale ranges are 0 to 2.5 for MK, Ka and Kr maps, and 0 to 3 × 10^− 3^ mm^2^/s for MD maps

All DKT imaging data were processed on an Advantage Windows Workstation (ADW 4.4 version, GE) with Functool software. The software performed a voxel-by-voxel analysis and could automatically output the values per voxel from each region of interest (ROI) measurements. DKTI metrics were automatically calculated and displayed on corresponding DKTI maps (Fig. 1).

For the diffusional kurtosis model, 3 b value data were fitted according to the following equation:

$$ \ln \left[S(b)/{S}_0\right]=-b{D}_{app}+\frac{1}{6}{b}^2{D}_{app}^2{K}_{app} $$

where *Dapp* represents the apparent diffusion coefficient, and *Kapp* represents the apparent kurtosis coefficient along a certain diffusion direction, *S*(*b*) is the diffusion weighted signal along that direction with a certain b value, and *S0* is the non-diffusion weighted signal. *Kapp* is a unitless parameter indicates the deviation of water motion from the Gaussian distribution. *Dapp* is a corrected ADC that removes this non-Gaussian bias.

Kurtosis metrics include mean kurtosis (MK), axial kurtosis (Ka), radial kurtosis (Kr). Ka and Kr are the kurtosis measured along the direction parallel and perpendicular to the principal diffusion direction, respectively. MK is the average kurtosis over all diffusion directions. Conventional diffusion metric (i.e. mean diffusity [MD]) was also derived from DKT imaging data.

Images were analyzed in consensus by two radiologists with 15 years’ experience (J, Z.) and 9 years’ experience (JY, G), respectively, who were unaware of the pathological results. Three non-overlapping oval or circular ROIs of 90mm^2^ ~ 110 mm^2^ were drawn on DKT images with b value of 0 s/mm^2^ on the lesions of kidney. According to the appearance on T2WI and T1WI, necrosis, cystic and bleeding region were carefully excluded. DKTI metrics of contralateral uninvolved renal parenchyma were measured by 2 readers separately to validate the inter-observer agreement. Three similar ROIs of 30 mm^2^ ~ 50 mm^2^ were placed at anterior, middle and posterior part of renal medulla on the central slice of uninvolved kidney. ROIs of renal cortex were drawn by hand with free curve with mean size of 150mm^2^ ~ 250 mm^2^. The values of DKTI metrics of 3 ROIs on renal lesions and renal medulla were averaged separately to represent those of renal lesions and renal medulla.

### Pathological assessment

All RCCs were confirmed by histopathological examination after total or partial nephrectomy. Histological subtypes were assigned for each lesion. The grading of CCRCC was assessed with the Fuhrman system, which stratified the tumor grade based on the size and shape of the nuclei and on the prominence of the nucleoli. All CCRCC were categorized into four grades (Grades I–IV) with grades I and II referred as low-grade tumor and grades III and IV as high-grade tumor.

### Statistical analysis

The DKTI metrics presented as mean ± standard deviation (SD) were tested firstly with the Kolmogorov-Smirnov test for normality and then with the Levene test for variance homogeneity.

First, inter-observer agreements in measurements of contralateral parenchyma were evaluated by using intraclass correlation coefficients (ICCs) (0–0.20, poor correlation; 0.21–0.40, fair correlation; 0.41–0.60, moderate correlation; 0.61–0.80, good correlation; and 0.81–1.00, excellent correlation). The reproducibility of DKTI metrics measurements was evaluated with the Bland-Altman method [18]. The mean absolute difference (bias) and the 95% confidence interval of the mean difference (limits of agreement [LOA]) between the measurements of two observers were calculated.

Statistical comparisons with DKTI metrics of 3 subtypes of RCC were performed with one-way analysis of variance (ANOVA) test. Multiple comparison was used with Scheffé test to show the difference between each pair of group. Data of all RCCs were also compared with those of contralateral renal parenchyma using paired- *t* test.

Finally, DKTI metrics of low grade and high grade CCRCC were compared with Student *t* test and receiver operating characteristic (ROC) curves were drawn to establish cut-off DKTI metrics. To compare the diagnostic value in differentiating different CCRCC tumor grades, the area under curve (AUC) was calculated (0.5–0.7, poor; 0.7–0.9, good; more than 0.9, excellent). The Spearman correlation was used to evaluate the association of all the DKTI metrics with histologic grades. The correlation coefficient (r) was obtained to compare the degree of correlation as follows: little if 0 < r <  0.25, fair if 0.25 < r <  0.5, moderate if 0.5 < r <  0.75, and excellent if r > 0.75.

Statistical analysis was performed by using SPSS (version 22.0; SPSS, Chicago, IL, USA) and MedCalc (version 19.0.4; MedCalc, Mariakerke, Belgium) software. A *P* value of less than 0.05 was considered as a significant difference.

## Results

### Patient demographics and histopathologic findings

In total, 38 patients (27 men, 11 women, mean age 60.1 years, age range 42–86 years) were involved in the study. Histopathologic analysis was performed on specimens acquired at radical (*n* = 30) and partial (*n* = 8) nephrectomy. The diameter of lesions were from 1.3 cm to 10.8 cm (median, 3.8 cm). Pathologic diagnoses of the 38 lesions included 30 CCRCC, 5 papillary RCC (PRCC) and 3 chromophobic RCC (CRCC). Of the 30 CCRCC, there were 22 low-grade and 8 high-grade CCRCC.

### Reproducibility of DKTI in contralateral renal parenchyma

Inter-observer measurements of DKTI metrics of contralateral renal cortex and medulla showed excellent agreement with ICCs ranging from 0.81 to 0.92, 95%CI 0.730 to 0.961.

**Fig. 2:**
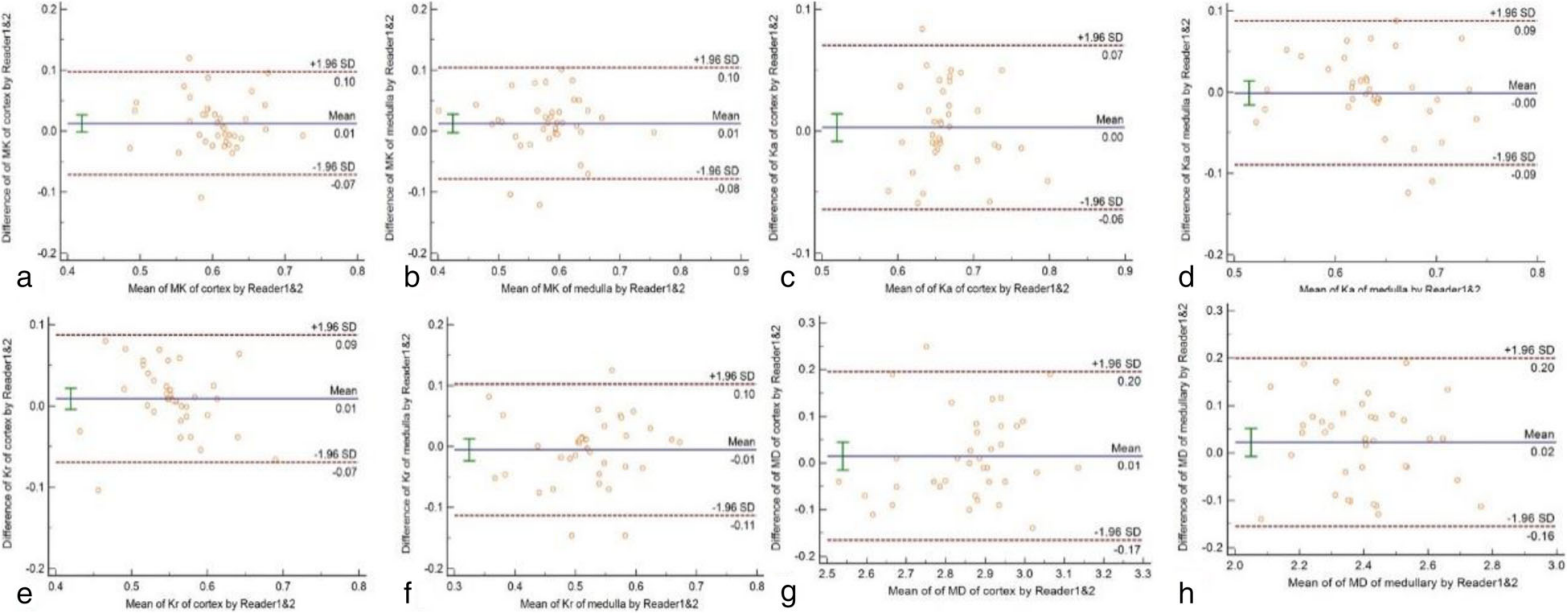
Bland-Altman plots show inter-observer agreement for MK, Ka, Kr and MD values of renal cortex (**a**, **c**, **e**, **g**) and medulla (**b**, **d**, **f**, **h**) of contralateral normal kidney

Mean biases of the measurement values by 2 readers with Bland-Altman method were shown in Fig. 2. The range of mean biases of MK, Ka, Kr and MD were from − 0.006 to 0.022, and LOAs were within 0.091.

### Comparison of DKTI metrics among RCC subtypes

Compared to the contralateral renal parenchyma with paired - *t* test, the values of MK, Ka, Kr of RCC were significantly higher than those of normal cortex and medulla, while the value of MD of RCC were significantly lower than those of normal parenchyma (Table 1). MK, Ka and Kr values of normal cortex were higher than medulla, but with no significant difference (*P* = 0.231, 0.369, 0.140, respectively). MD values of normal cortex were significantly lower than those of medulla (*P* <  0.001).

**Table 1:** Comparison of DKTI metrics (mean ± SD) between RCC and contralateral renal cortex and medulla

DKTI metrics	RCC	Cortex	***P*** value	Medulla	***P*** value
**MK**	0.77 ± 0.23	0.61 ± 0.06	< 0.001	0.59 ± 0.07	0.001
**Ka**	0.86 ± 0.27	0.67 ± 0.05	0.005	0.64 ± 0.06	0.001
**Kr**	0.64 ± 0.21	0.56 ± 0.06	0.003	0.52 ± 0.09	0.002
**MD (10**^**−3**^ **mm**^**2**^**/s)**	1.61 ± 0.43	2.87 ± 0.15	< 0.001	2.41 ± 0.16	< 0.001

There were also significant difference of DKTI metrics among 3 subtypes of RCC performed with ANOVA test (Table 2, Fig. 3). Post Hoc Scheffé Test showed that MK and Ka values of CRCC were significant higher than those of CCRCC and PRCC.

**Table 2:** Comparison of DKTI metrics (mean ± SD) of RCC subtypes

DKTI metrics	CCRCC (30 cases)	CRCC (3 cases)	PRCC (5 cases)	***P*** value
**MK**	0.74 ± 0.19	1.27 ± 0.11^a^	0.62 ± 0.06	< 0.001
**Ka**	0.83 ± 0.23	1.39 ± 0.08^b^	0.70 ± 0.20	< 0.001
**Kr**	0.63 ± 0.19	0.97 ± 0.15	0.50 ± 0.10	0.006
**MD (10**^**−3**^ **mm**^**2**^**/s)**	1.68 ± 0.41	0.97 ± 0.07	1.58 ± 0.43	0.023

#1 *CCRCC* clear cell RCC, *CRCC* chromophobic RCC, *PRCC* papillary RCC

#2 ^a,b^: Post Hoc Scheffé Test of multiple comparison of each two pair of RCC subtypes for all metrics showed that the MK and Ka values of CRCC were significantly higher than those of CCRCC and PRCC with *P* values < 0.001 (*P* < 0.017 was considered significant with Bonferroni correction)

**Fig. 3:**
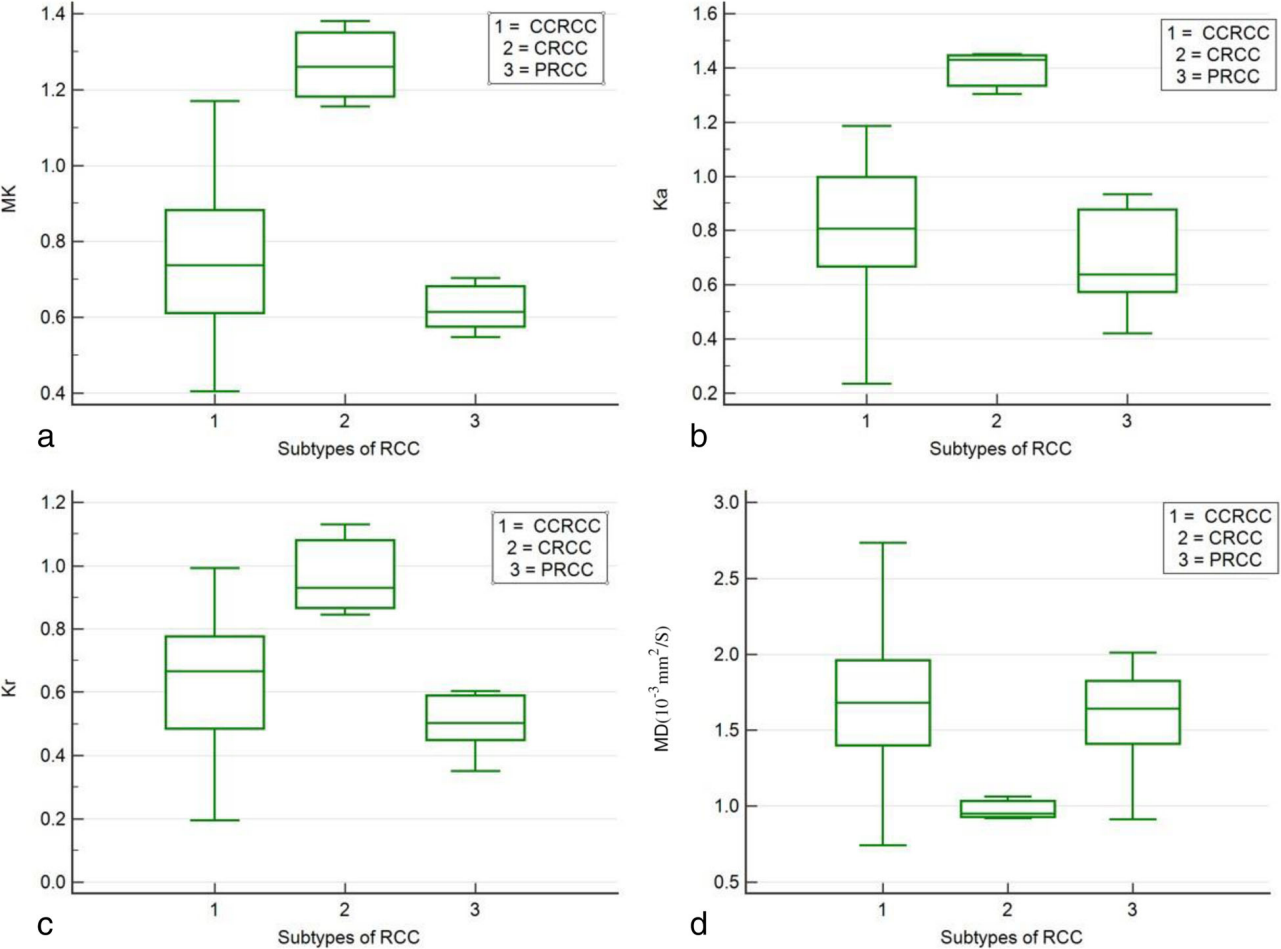
Box-and-whisker plot distribution of MK (**a**), Ka (**b**), Kr (**c**) and MD (**d**) values for 3 subtypes of RCC. The top and bottom of each box represent the 25 and 75% percentiles, respectively, of the metrics. The horizontal line inside each box represents the median value

### Comparison of DKTI metrics between low-grade and high-grade CCRCC

**Fig. 4:**
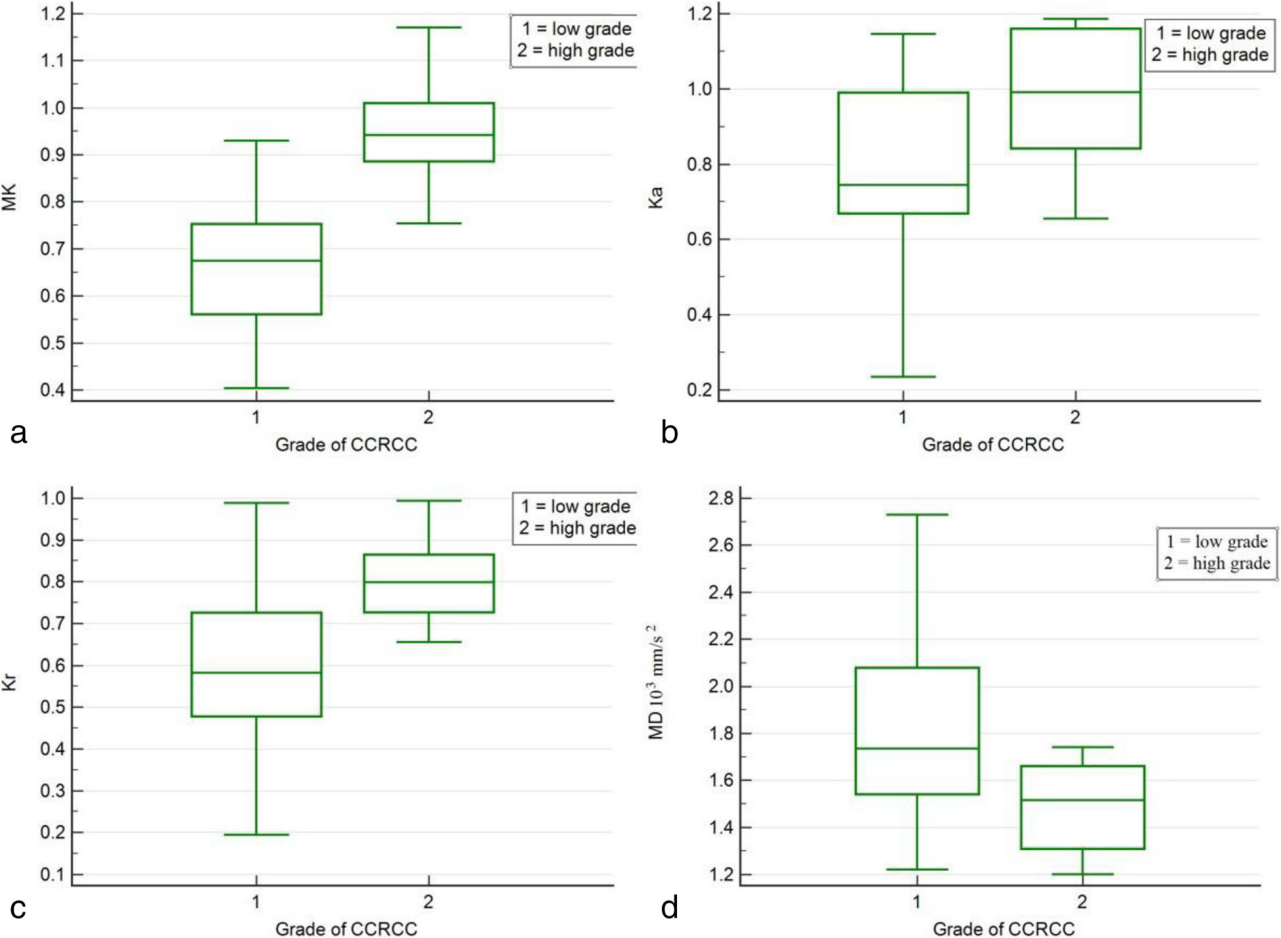
Box-and-whisker plot distribution of MK (**a**), Ka (**b**), Kr (**c**) and MD (**d**) values for low- and high- grade CCRCC. The top and bottom of each box represent the 25 and 75% percentiles, respectively, of the metrics. The horizontal line inside each box represents the median value

**Fig. 5:**
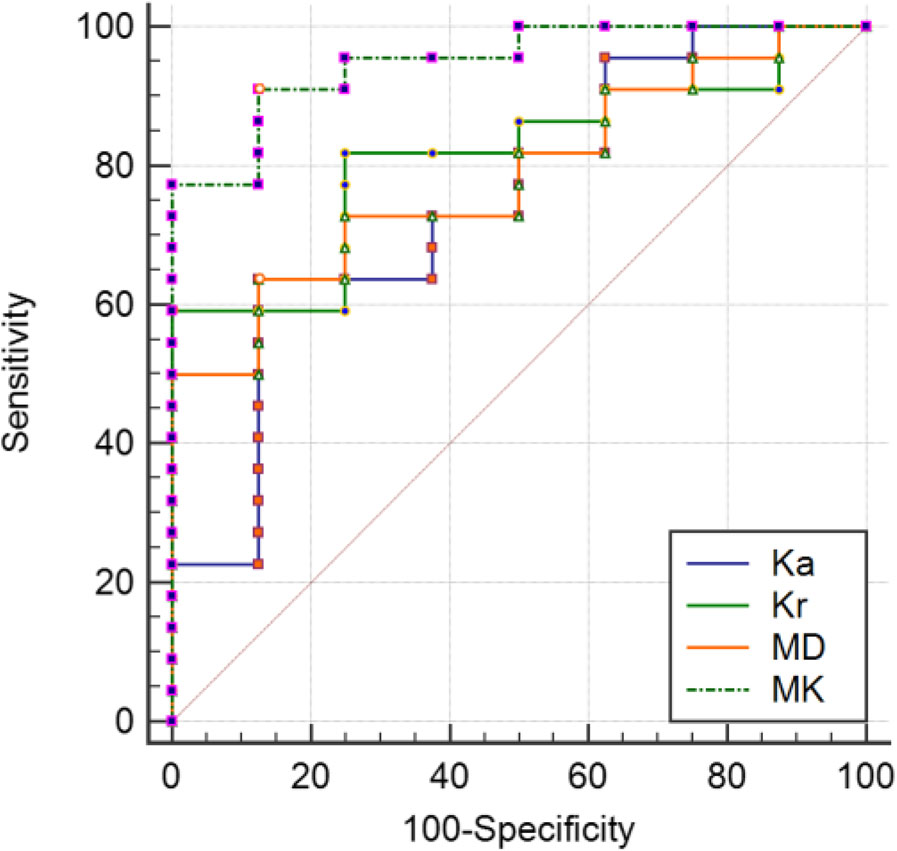
The ROC curves was performed to differentiate low-grade and high-grade CCRCC using MK (AUC = 0.949), Ka (AUC = 0.750), Kr (AUC = 0.812) and MD values (AUC = 0.784)

Statistical difference of MK, Ka, Kr and MD values were also obtained among 30 cases of CCRCC with low- and high-grade compared with Student *t* test (Table 3, Fig. 4). MK, Ka and Kr values of low- grade CCRCC were significantly lower than those of high-grade CCRCC (*P* < 0.05), whereas MD value of low-grade CCRCC was significantly higher than that of high-grade CCRCC (*P* = 0.023). The AUC of MK, Ka, Kr and MD for the assessment of low- and high-grade CCRCC were 0.949 (95% CI: 0.801–0.996, *P* < 0.001), 0.750 (95% CI: 0.559–0.889, *P* = 0.018), 0.813 (95% CI: 0.628–0.931, *P* < 0.001), and 0.784 (95% CI: 0.596–0.912, *P* < 0.001) respectively (Fig. 5). AUC of MK was significantly higher than that of Ka, Kr and MD with *P* value of 0.039, 0.022 and 0.0425, respectively). The optimal cutoff value of MK was 0.860 with sensitivity of 90.9% and specificity of 87.5%.

**Table 3:** Comparison of DKTI metrics (mean ± SD) of low grade and high grade clear cell carcinoma

DKTI metrics	Low grade (22 cases)	High grade (8 cases)	***P*** value
**MK**	0.66 ± 0.14	0.95 ± 0.12	0.000
**Ka**	0.78 ± 0.22	0.99 ± 0.18	0.018
**Kr**	0.57 ± 0.19	0.80 ± 0.11	0.003
**MD (10**^**−3**^ **mm**^**2**^**/s)**	1.81 ± 0.36	1.48 ± 0.2	0.023

MK exhibited a significantly moderate positive correlation with the Furhman grades of CCRCC (*r* = 0.688, *P* < 0.001), whereas Ka, Kr and MD showed fair positive correlations with the Furhman grades (Ka: *r* = 0.383, *P* = 0.036, Kr: *r* = 0.479, *P* = 0.007, MD: *r* = 0.435, *P* = 0.016).

## Discussion

This study explored the reproducibility and feasibility of diffusion kurtosis tensor imaging, a non-Gaussian diffusion model, in the assessment of RCC with defining the histological subtype and the grades of the tumor.

DKTI model was initially applied in the brain to quantify the tissue heterogeneity of microstructure by measuring the deviation of the signal curve from monoexponential DTI at high b values (the maximum b value of 2000 s/mm^2^ were recommended [8]). Due to the faster T2 decay property and the respiratory motion, body tissue suffers from lower signal-to-noise ratio (SNR) with strong b values compared to brain. The b values were 0, 500, 1000 s/mm^2^ in our research which were similar with previous studies performing DKTI on kidney [12, 19, 20]. In this range of b values, the total signal decay was influenced by blood perfusion in microvascular network in addition to the pure diffusive effects. Perfusion affected DKI through the intravoxal incoherent motion (IVIM) mechanism [21–23]. De Luca et al. [22] found positive correlation of MK value and f value from IVIM measuring the fractional volume of capillary blood in the skeleton muscle. Minosse et al. [24] showed positive association between MK and En(Kep) from dynamic contrast MR in head and neck cancer, which suggested that MK might link to the tumor vascular heterogeneity and complexity represented by En (Kep). The effect of perfusion-related signal decay on DKI metric estimates should be taken into account when DKI model performed with a minimum b-value less than 300 s/mm^2^ [21].

In our study, to validate the reproducibility of DKTI in kidney, both of the cortex and medulla of contralateral normal kidney were measured because of the difference in anatomy and histological structure between cortex and medulla. The previous studies also showed the corticomedullary differentiation detected by DKTI [12, 19]. As our results showed, DKTI parameters of the contralateral cortex and medulla measured by 2 observers had excellent correlation with ICC values from 0.81 to 0.92, and there was good inter-observer agreement of measurements of DKTI, which was consistent with the results in previous study [19]. MK, Ka, and Kr values of RCC were significantly higher than those of contralateral renal cortex and medullary, and vice versa for MD. It indicated that DKTI could readily discriminate normal renal parenchyma from RCC with good reproducibility.

Most of the previous studies on the histological characteristics of RCC were mainly performed by DWI. ADC values based on DWI showed moderate performance to differentiate between high- and low-grade CCRCC [25], but the ADC values of the 3 most common subtypes of RCC reported by different research groups were not coherent [3, 26, 27]. This issue probably arose from the variances in the magnetic fields, vendor of MR systems, or the parameters of DWI sequence, especially with b values. Although the results are controversy, the pattern that higher ADC values are associated with CCRCC seems unambiguous. More recently, other methods, such as IVIM, DTI and DKI, were applied as the extension of conventional DWI to give more information on the assessment of histopathological findings of RCC [13, 28, 29].

Our results showed that MK and Ka values of CRCC were significantly higher than those of CCRCC and PRCC. As patients with CRCC has better prognosis than those with CCRCC and PRCC [30], distinguishing CRCC from the other 2 common subtypes of RCC before surgery is essential for choosing an appropriate strategy and predicting the prognosis. Zhang et al. [31] and Ding et al. [28] found that MK value of CCRCC (0.62, 0.68, respectively) was lower than non-CCRCC (0.9, 0.88, respectively). As they all include CRCC and PRCC as one group (non-CCRCC), it could not be determined whether CRCC owed highest MK values among 3 subtypes of RCC. However, in our study, MK value of CCRCC was not lower than non-CCRCC. The parameters of DKI sequence such as b values and number of diffusion directions were different between our study and research of Zhang et al. and Ding et al.. Multicenter, large scale trials with standardized acquisition protocols are needed to verify the characteristic of DKI of subtypes of RCC. An exact explanation for the higher kurtosis values of CRCC than those of CCRCC and PRCC is not easy. In our research, the increased perfusion and restricted diffusion properties were characterized simultaneously. Some features of microscopic structure of CRCC might contribute to the complex cellularity architecture, including the mixture of two kinds of cells (eosinophilic and clear cytoplasm) in various size with narrow cell gap, dense cytoplasm, perinuclear halos and distinct cell membranes. Whereas, the studies on the perfusion of subtypes of RCC demonstrated that perfusion of CRCC and CCRCC quantified by f value (IVIM) and renal blood flow (mL/min/100 g) (Arterial Spin Labeling, ASL) were significantly higher than those of PRCC, with no difference between those of CRCC and CCRCC [32, 33]. Hempel et al. [23] demonstrated that perfusion-corrected MK performed slightly poorly than perfusion-biased MK in classification of glioma, which indicated that taking the effect of perfusion on DKI metrics into account did not substantially impact their overall diagnostic performance in classifying glioma. The relationship between restricted water diffusion and microvascular perfusion remains unclear, and the mechanisms underlying the differences in the alterations of kurtosis metrics with subtypes of RCC have yet to be determined.

Fuhrman grade is one of the most well-known prognostic factors which is associated with cancer-specific survival after nephrectomy [34]. In our study, MK, Ka, Kr values of high-grade CCRCC were significantly higher than those of low- grade CCRCC, and vice versa for MD, which was consistent with previous studies [13, 35]. We also found that MK values demonstrated higher diagnostic performance than MD for determination of CCRCC gradings, which supported by the researches of Dai et al. [13] and Wu et al. [17]. Compared to diffusion metrics, MK can be more robust to assess the tumor cellularity and vascularity in CCRCC with different grades. Histopathologicallly, restricted water diffusion and increased tissue perfusion become more prominent in higher tumor grade of CCRCC. High-grade CCRCC were characterized by higher cellularity, more nuclear atypia, higher pleomorphism, and more vascular hyperplasia and necrosis [36], which lead to increased complexity of the intracellular microenvironment. Perfusion in high-grade CCRCC are also increased with predominant blood volume and tumor vascularization, verified by the studies performed with IVIM [31, 37] and dynamic contrast MR [38]. MK can be a surrogate biomarker for predicting nuclear grade of CCRCC.

Our study had some limitations. First, in our research, DKTI model address both diffusion and perfusion behavior of RCC and it was hard to find out whether the main deviation from a monoexponeatial curve occurred at the low-b range or at the high-b range. An extensional model combining DKTI and IVIM (DKTI-IVIM) [39, 40] should be introduced to measure diffusion and perfusion effect separately. More b values in suitable intervals with larger scale of b values should be taken to compare the results from an IVIM analysis (with the range of b-values from 0 to 700 s/mm^2^) and DKI analysis (with the range of b-values from 300 to 1500 s/mm^2^). Second, as rare histological types, the sample size of PRCC and CRCC were less compared to CCRCC. Larger patient populations with PRCC and CRCC are needed to confirm the result. Third, the regions of interest were selected in the solid compartment of tumor instead of the entire tumors in this study, which might lead to some position-dependent bias owing to tumor heterogeneity. Histogram analysis, which had shown value in providing more quantitative information about tumor heterogeneity in other studies of RCC [35], were not assessed.

## Conclusion

DKTI showed good feasibility and reproducibility in detecting the histologic characteristics of RCC. MK, the principle parameter of DKTI, may be an additional biomarker in detection of subtypes of RCC and in active surveillance by assessing histologic grade.

## Data Availability

The datasets used and/or analysed during the current study are available from the corresponding author on reasonable request.

## Abbreviations

ADC: Apparent diffusion coefficient
ASL: Arterial Spin Labeling
AUC: Area under curve
CCRCC: Clear cell renal cell carcinoma
CI: Confidence interval
CRCC: Chromophobic renal cell carcinoma
DKI: Diffusion kurtosis imaging
DKTI: Diffusion kurtosis tensor imaging
DWI: Diffusion-weighted imaging
ICC: Interclass correlation coefficient
Ka: Axial kurtosis
Kr: Radial kurtosis
MK: Mean kurtosis
MD: Mean diffusity
PRCC: Papillary renal cell carcinoma
RCC: Renal cell carcinoma
ROC: Receiver operating characteristic
ROI: Region of interest
TE: Echo time
TR: Repetition time
